# An intestinal model with a finger-like villus structure fabricated using a bioprinting process and collagen/SIS-based cell-laden bioink

**DOI:** 10.7150/thno.41225

**Published:** 2020-01-22

**Authors:** WonJin Kim, Geun Hyung Kim

**Affiliations:** Department of Biomechatronic Engineering College of Biotechnology and Bioengineering Sungkyunkwan University (SKKU), Suwon 16419, South Korea.

## Abstract

The surface of the small intestine has a finger-like microscale villus structure, which provides a large surface area to realize efficient digestion and absorption. However, the fabrication of a villus structure using a cell-laden bioink containing a decellularized small intestine submucosa, SIS, which can induce significant cellular activities, has not been attempted owing to the limited mechanical stiffness, which sustains the complex projective finger-like 3D structure. In this work, we developed a human intestinal villi model with an innovative bioprinting process using a collagen/SIS cell-laden bioink.

**Methods**: A Caco-2-laden microscale villus structure (geometry of the villus: height = 831.1 ± 36.2 *μ*m and diameter = 190.9 ± 3.9 *μ*m) using a bioink consisting of collagen type-I and SIS was generated using a vertically moving 3D bioprinting process. By manipulating various compositions of dECM and a crosslinking agent in the bioink and the processing factors (printing speed, printing time, and pneumatic pressure), the villus structure was achieved.

**Results**: The epithelial cell-laden collagen/SIS villi showed significant cell proliferation (1.2-fold) and demonstrated meaningful results for the various cellular activities, such as the expression of tight-junction proteins (ZO-1 and E-cadherin), ALP and ANPEP activities, MUC17 expression, and the permeability coefficient and the glucose uptake ability, compared with the pure 3D collagen villus structure.

**Conclusion**: *In vitro* cellular activities demonstrated that the proposed cell-laden collagen/dECM villus structure generates a more meaningful epithelium layer mimicking the intestinal structure, compared with the pure cell-laden collagen villus structure having a similar villus geometry. Based on the results, we believe that this dECM-based 3D villus model will be helpful in obtaining a more realistic physiological small-intestine model.

## Introduction

The epithelium of the small intestine consists of a single layer of cells and a topographical structure of microscale projective villi, whose height ranges from several hundred micrometers to a few millimeters. This layer provides the large surface needed to induce effective digestion of food, absorption of nutrients, and waste excretion 1,2. However, the fabrication of a realistic 3D scaffold mimicking the structure on the intestine surface for measuring *in vitro* drug permeability and for regenerating the small intestinal tissue is still being investigated 3-5.

Conventionally, a two dimensional (2D) epithelial monolayer model has been used for assessing intestinal diseases, drug development, and nutrient absorption ability 5, 6. However, although the 2D flat structure could adequately support the complex metabolism of epithelial cells and bacteria, this simplified 2D intestinal model cannot fully mimic realistic absorption kinetics and terminal differentiation, owing to the absence of a geometrical crypt-villus structure, resulting in insufficient replication of the function of human pathophysiology 4, 5. To address the topographical issue of the small intestine, more complex 3D intestinal models have been developed using microfabrication methods, such as laser ablation/sacrificial molding 7, replicating molds 8, and direct printing 9, because the finger-like 3D villus structure could directly influence the intestinal flow, pressure, and surface stiffness, eventually affecting the cell morphology and biochemical properties of the cultured epithelial cells (Table 1) 4, 7-9. Furthermore, many attempts have utilized a hydrogel consisting of natural polymers instead of synthetic polymers, such as poly(lactic-co-glycolic acid) (PLGA), and polydimethylsiloxane (PDMS), to overcome the physiological limitations 4, 7 ,9. For example, a 3D microsized gastrointestinal tract was fabricated using a sacrificial alginate mold and collagen hydrogel 4. Using the finger-like structure, Caco-2 cells were cultured, and various cellular and physiological activities were evaluated. According to the results, the 3D villus structure showed a significantly higher MUC17 expression compared with a 2D monolayer model. In addition, a 3D microfluidic model using a PDMS membrane showed a significant uptake efficiency of glucose, higher than that of a monolayer model 10. While these models have successfully fabricated complex topography of finger-like structure using extracellular matrix (ECM)-based hydrogels, but further studies on the biological support is still needed.

Decellularized biomaterials have attracted attention because they possess various biocomponents derived from native complex tissues and vasculature 11-15. Recently, a decellularized structure was used as a natural intestinal scaffold because it contained the natural intestinal ECM structure and connective tissue biofactors 16. According to the results of the study, the decellularized intestine scaffold supported a significantly high degree of intestine regeneration owing to the maintenance of the ECM structure and angiogenic components. The small intestinal submucosa (SIS) was a decellularized ECM of the submucosal layer of a porcine small intestine, and the biodegradable material consisted of collagen types I and III, glycosaminoglycans, and various growth factors, such as the transforming growth factor, basic fibroblast growth factor, connective tissue growth factor, vascular endothelial growth factor, and so on 17,18. Owing to the various bioactive components of the SIS and its continuous ability to repair damaged tissues 19, it has been widely applied in regeneration of various tissues, such as the skin 20, cardiovascular 21, ligament 22, gastrointestinal 23, and corneal tissues 24. In addition, the genipin-crosslinked SIS has been used to regenerate gastric mucosa, and it induces the acceleration of defected mucosa regeneration 25.

In this study, a collagen/SIS-based Caco-2-laden biomimetic 3D villus structure was fabricated for observing the formation of intestinal biological structure using *in vitro* culture. To obtain an array of cell-laden finger-like microstructures with an analogous intruding shape and similar dimensions to those of the human villus (height: 200-1000 μm, diameter: 100-200 μm, density: 20-40 villi/mm^2^ 26, 27), we employed a 3D bioprinting process 9, 28, 29. To improve the printability and biodegradability of the collagen/SIS hydrogel, tannic acid (TA), as a crosslinking agent, was used. In addition, to obtain a crypt-villus geometry similar to that of the real human intestine, the following two-step process was applied: first, a single layer was printed as a mesh structure using a conventional 3D printing process 30-33, and second, several villi were vertically printed on the crossed region of the mesh structure. To achieve the 3D villus structure laden with collagen/SIS and Caco-2 cells, which is designed to have a protruding shape and high aspect ratio (height/diameter = ~5) and induce a considerable level of cellular activity, several processing factors, including the concentration of SIS, weight fraction of the crosslinking agent, vertical printing speed, and pneumatic pressure, were employed. After printing the structure, various cellular activities, such as cell-viability, proliferation, junction markers (E-Cadherin and ZO-1), alkaline phosphatase (ALP) activities, alanyl (membrane) aminopeptidase (ANPEP) activities, and gene expression of MUC17 (one of the transmembrane mucins), of the cultured intestinal epithelial cells were observed.

## Experimental Section

### Preparation of decellularized small intestinal submucosa powder

The decellularization procedure for the porcine SIS was performed based on a previously reported method 34. Briefly, porcine jejunum sections, harvested from an adult Yorkshire porcine (female, 10-15 months old) immediately after sacrifice, were carefully washed with deionized water (DW). The fat was removed, and the tissue sections were cut into pieces (~10 cm in length). Then, the tunica serosa and tunica muscularis layers were completely removed mechanically using a Petri dish, to obtain the SIS, followed by washing with phosphate-buffered saline (PBS; pH = 7.0). The SIS was incubated with 0.1% (w/v) peracetic acid (Sigma-Aldrich, USA) to remove the cellular remnants and washed in PBS and DW. Following lyophilization using a freeze-dryer (SFDSM06; Samwon, South Korea), the decellularized SIS was ground using a cryogenic grinder (6800 Freezer/Mill^®^; SPEX SamplePrep, USA)) to obtain SIS powder. The SIS powder was stored at -80 °C until use.

### Characterization of decellularized SIS

For visualizing the morphology of native SIS and decellularized SIS powder, the samples were lyophilized and observed using a scanning electron microscope (SEM) (SNE-3000M, SEC Inc., South Korea) and an optical microscope (BX FM-32; Olympus, Japan).

To verify the decellularization, the native and decellularized (before grinding) SIS were fixed with 3.7% formaldehyde (Sigma-Aldrich, USA) at 37 °C for 1 day. For the quantification of the DNA, collagen, glucoseaminoglycans (GAGs), and elastin contents, the dehydrated tissues were freeze-dried. The quantification analyses were performed on 20 mg of each of the dry tissues using a Quant-iT PicoGreen dsDNA Assay kit (ThermoFisher Scientific) and Sircol^TM^ Soluble Collagen, Blycan^TM^ Sulfated Glycosaminoglycans, and Fastin^TM^ Elastin assay kits (Biocolor Life Sciences Assays, UK) according to the protocols of the manufacturer.

To confirm the remaining ECM after the decellularization process, the collagen type I and elastin of the tissues were visualized. The fixed tissues were treated with a bovine serum albumin (BSA; Sigma-Aldrich, USA) for 2 h and permeabilized using 2% Triton X-100 for 30 min. The permeabilized tissues were then incubated with an anti-collagen-I primary antibody (5 μg/mL in PBS; Invitrogen, USA) and an anti-elastin primary antibody (5 μg/mL in PBS; Invitrogen, USA) overnight at 4 ºC. The primary antibody-treated samples were rinsed with PBS and stained with an Alexa Fluor 594 (1:50 in PBS; Invitrogen, USA) conjugated secondary antibody for 1 h at 37 °C, followed by counterstaining of the nuclei with 5-μM diamidino-2-phenylindole (DAPI; Invitrogen, USA). A Carl Zeiss confocal microscope (LSM 700; Carl Zeiss, Germany) was used to visualize the collagen and elastin of the native and decellularized SIS.

### Preparation of collagen/SIS bioink

For the collagen/SIS bioink, Collagen-I from porcine skin (MSBio, South Korea) and decellularized SIS powder were mixed in 0.1-M acetic acid, and mixed with Dulbecco's modified Eagle's medium, ×10 enriched (×10 DMEM; Sigma-Aldrich) solution 35. The final concentration of collagen (4 wt%) was fixed, whereas different concentrations (0, 10, 20, 30, and 40 mg/mL) of decellularized SIS powder were used. The neutralized bioinks were then mixed with the Caco-2 cells (5 × 10^6^ cells/mL; American Type culture Collection, USA). Various concentrations (0, 1, 2, 3, and 4 wt% in PBS) of TA (Sigma-Aldrich, USA), the crosslinking agents of collagen, were mixed with the bioinks.

As a control, neutralized collagen (4 wt%) bioink mixed with the Caco-2 cells (5 × 10^6^ cells/mL) and TA (2 wt%) was used (collagen bioink).

### Fabrication of Caco-2-laden 3D intestinal villi

The Caco-2-laden 3D intestinal villi with collagen/SIS bioink (CLIV-CS) were fabricated using a three-axis printing system (DTR3-2210 T-SG; DASA Robot, South Korea). The collagen/SIS bioink was printed onto the 37 °C working plate through a 25G single nozzle (inner diam.: 250 μm) using a pneumatic pressure dispensing system (DTR2-2210T; Dongbu Robot, South Korea). To obtain the 3D model, following the printing of a crypt section as a layer of a 0°/90° strut (mesh) structure using fixed printing conditions (pneumatic pressure of 275 kPa and moving speed of 10 mm/s), the villus structures were then fabricated onto the mesh structure by using a vertically moved 3D printing (VMP) process. For printing each villus structure, the applied pneumatic pressure (275 kPa) and raising speed (5 mm/s) were fixed for 0.5 s of extrusion time. The fabricated 3D structure was then immersed in minimum essential medium (MEM; Gibco^TM^, USA) and PBS.

As a control, the collagen bioink was used to obtain the 3D model (CLIV-C). The fabrication conditions were the same as those of the CLIV-CS, except for the applied pneumatic pressure (250 kPa).

The same fabricating conditions for printing the crypt structures was used to obtain cell-laden mesh structures without villus structures using collagen bioinks (CLM-C) and collagen/SIS bioink (CLM-CS).

To obtain the 3D intestinal villi containing an epithelium layer and capillaries, we used two bioinks; (i) Caco-2-laden collagen/SIS bioink (shell bioink) and (ii) human umbilical vein endothelial cell (HUVEC)-laden collagen/SIS bioink (core bioink). The bioinks were printed using a three-axis printing system connected with core/shell nozzle (core inner dia.: 250 μm; shell thickness: 180 μm; NanoNC, South Korea). The fabrication conditions were the same as those of the CLIV-CS, except for the applied pneumatic pressure. For printing each villus structure, the pneumatic pressures were simultaneously applied for the core region (core bioink; 275 kPa) and the shell region (shell bioink; 125 kPa.

### Characterization of the bioink and Caco-2-laden 3D intestinal villi

The rheological properties, such as storage modulus (G′), loss modulus (G''), complex viscosity (η^∗^), and yield stress (σ_y_), of the collagen/SIS bioinks with diverse concentrations of decellularized SIS powder (0, 10, 20, 30, and 40 mg/mL) were evaluated using a rotational rheometer (Bohlin Gemini HR Nano; Malvern Instruments, UK) supplemented with a cone-and-plate geometry (cone angle: 4°, diameter: 40 mm, gap: 150 μm). G′ and η^∗^ were obtained by conducting a frequency sweep (from 0.1 Hz to 10 Hz) within the linear viscoelastic region with 1% strain at 25 °C. To analyze the yield stress of the bioinks, G' and G'' were evaluated by conducting a stress sweep (from 10 Pa to 500 Pa) with 1 Hz at 25 °C.

The SEM and optical microscope were used to visualize and characterize the surface morphology of the Caco-2-laden 3D intestinal models. The obtained optical and SEM images were used to calculate the height, diameter, and aspect ratio of the villus structure.

### *In vitro* cell culture

The MEM, containing penicillin/streptomycin (PS) (1%; Caisson Labs, USA), fetal bovine serum (FBS) (10%; Gemini Bio-Products, USA), 4-(2-hydroxyethyl)-1-piperazineethanesulfonic acid (HEPES) (25 mM; Sigma-Aldrich, USA), and sodium bicarbonate (25 mM; Sigma-Aldrich, USA), was used to culture the fabricated cell-laden villus models. The medium was changed every 2 days.

### *In vitro* cellular activities

The proliferation of Caco-2 cells in the 3D models was evaluated by measuring the DNA contents using the Quant-iT PicoGreen dsDNA Assay kit, according to the protocols of the manufacturer. Briefly, the 3D intestinal models were treated with a cell lysis buffer containing ethylenediaminetetraacetic acid (EDTA) (1 mM; Sigma-Aldrich, USA), Tris-HCL (10 mM; Sigma-Aldrich, USA), and Triton X-100 (0.2 v/v%; Sigma-Aldrich, USA) for 30 min. The cell lysate was assayed with the Quant-iT PicoGreen^®^ reagent, and the fluorescence of the dye was measured using a synergy H1 Hybrid Reader (BioTek Instruments; excitation = 480 nm, emission = 520 nm).

To observe the live and dead cells, the Caco-2 cells in the bioinks and fabricated 3D model were stained with calcein acetoxymethyl ester (calcein AM) (0.15 mM; Invitrogen, USA) and ethidium homodimer-1 (EthD-1) (2 mM; Invitrogen, USA) at 37 °C for 1 h. A confocal microscope (LSM700; Carl Zeiss, Germany) was used to obtain images of the stained live (green) and dead (red) cells. The cell viability was evaluated using the ImageJ software.

The Caco-2 cells in the printed 3D models were stained using diamidino-2-phenylindole (DAPI) (1:100 in DPBS; Invitrogen, USA) and Alexa Fluor 568 conjugated phalloidin (1:100 in DPBS; Invitrogen) to visualize the nuclei and cytoskeleton of the cells. The confocal microscope was used to observe the stained nuclei (blue) and cytoskeleton (red) of the cells. Using the ImageJ software, the cell coverage rate was evaluated.

### Alkaline phosphate (ALP) and Aminopeptidase N (ANPEP) Activities

ALP and ANPEP activities, differentiation markers of the matured enterocytes of the Caco-2 cells in the 3D models, were evaluated based on the previously described methods 36, 37.

The ALP activity was evaluated by measuring *p*-NP. Briefly, the samples were treated with Tris buffer (10 mM, pH 7.5) containing Triton X-100 (0.1 v/v%). The cell lysate and *p*-nitrophenyl phosphate (*p*-NPP) were placed on 96-well microplates to activate the enzymatic activity, and NaOH solution (0.5 M) was added to stop the activity. The level of ALP activity was measured by using a SpectraIII microplate reader (SLT Lab Instruments) with absorbance at 405 nm and normalized to total DNA contents. All data values were presented as mean ± standard deviation (SD) (n = 6).

For evaluating the ANPEP activity, *p*-nitroanilide (*p*-NA) was measured. The 3D models were incubated in a reaction buffer containing _L_-Ala-NA (5 mM), Tris-HCL (10 mM) and NaCl (150 mM), and the ANPEP activity level was measured by using the SpectraIII microplate reader with absorbance at 405 nm and normalized to total DNA contents. All data values were presented as mean ± SD (n = 6).

### Cell-tracker

To visualize the cell distribution after printing, the cells were stained with CellTracker (Molecular probes, USA) according to the protocol of the manufacturer before mixing cells with the prepared collagen/SIS hydrogels. Caco-2 cells and HUVECs were harvested using trypsin/EDTA solution (Gibco^TM^, USA) and incubation in CellTracker solution (37 °C) for 30 min. After removing CellTracker solution, the stained cells (Caco-2, red and HUVEC, green) were mixed with each collagen/SIS hydrogels. The cells were visualized using the confocal microscope.

### Immunofluorescence

Before the immunofluorescence analysis, the cultured 3D cell-laden models were washed with PBS. The specimens were incubated in 10% formalin for 60 min at room temperature. The fixed samples were then blocked with a bovine serum albumin (BSA) (2 wt%; Sigma-Aldrich, USA) for 2 h at 37 °C, and permeabilized with Triton X-100 (2 v/v%; Sigma-Aldrich, USA) for 30 min at 37 °C. Then, the 3D models were treated with an anti-MUC17 primary antibody (5 μg mL^-1^; Developmental Studies Hybridoma Bank, USA), anti-E-cadherin primary antibody (5 μg mL^-1^; Invitrogen, USA), anti-ZO-1 primary antibody (5 μg mL^-1^; Invitrogen, USA), anti-Laminin primary antibody (5 μg mL^-1^; Invitrogen, USA), and CD31 (5 μg mL^-1^; Abcam, USA) overnight at 4 °C. Subsequently, the primary-antibody-treated 3D models were rinsed with PBS and stained with Alexa Fluor 488 conjugated secondary antibody (1:50 in PBS; Invitrogen, USA) and Alexa Fluor 568 conjugated secondary antibody (1:50 in PBS; Invitrogen, USA) for 1 h. The stained structures were counterstained with DAPI (Invitrogen, USA). The immunofluorescence images of the 3D models were captured using the confocal microscope. To measure the area of MUC17, ZO-1, and E-cadherin, the ImageJ software was used.

### Functionality of the 3D models

The barrier integrity of the epithelial models (CLIV-C, and CLIV-CS) was evaluated by measuring the permeability coefficient and the glucose uptake ability using a Transwell based on previously described protocols 36.

To measure the permeability, CLIV-C and CLIV-CS were rinsed using Hank's balanced salt solution (HBSS) (Sigma-Aldrich, USA) and placed on the apical (AP) region of the Transwell. Fluorescein isothiocyanate-dextran (FITC-dextran, 4 kDa) (5 mg/mL in HBSS; Sigma-Aldrich) was added to the AP region, and HBSS was added to the basolateral (BL) part. After incubation for 1 h at 37 °C, the fluorescence of the solution in the BL chamber was measured using the synergy H1 Hybrid Reader (excitation = 492 nm, emission = 518 nm). The permeability coefficient values were calculated using the known concentrations of FITC-dextran.

The glucose uptake ability of the 3D models was quantified using an Amplex Red glucose assay kit (Molecular probes, USA). To remove the glucose, the 3D intestinal models were preincubated in PBS+ consisting of CaCl_2_ (1 mM) and MgCl_2_ (1 mM) at 37 °C and were placed on the AP part of the Transwell. Glucose (30 μM in PBS+) was added to the AP chamber whereas PBS+ was added to the BL part. An aqueous solution in the BL chamber was transferred into a 96-well microplate every 10 min, followed by incubation with a working solution containing Amplex Red reagent (100 μM), horseradish peroxidase (HRP; 0.2 U mL^-1^), and glucose oxidase (2 U mL^-1^), at room temperature for 30 min. The synergy H1 Hybrid Reader (excitation = 530 nm, emission = 590 nm) was used to measure the glucose transportation amount. All data values were presented as mean ± SD (n = 5).

### Statistical analyses

To perform the statistical analyses, the SPSS software (SPSS, Inc., USA) was used. A T-test was performed on the comparison between native *vs*. decellularized SIS, and CLIV-C *vs*. CLIV-CS, and a single-factor analysis of variance (ANOVA) and Turkey's honestly significant difference (HSD) test were performed on the other statistical analyses (^*^P < 0.05 was considered statistically significant).

## Results and discussion

### Fabrication of SIS and characterization of collagen-SIS bioink

**Figure 1:**
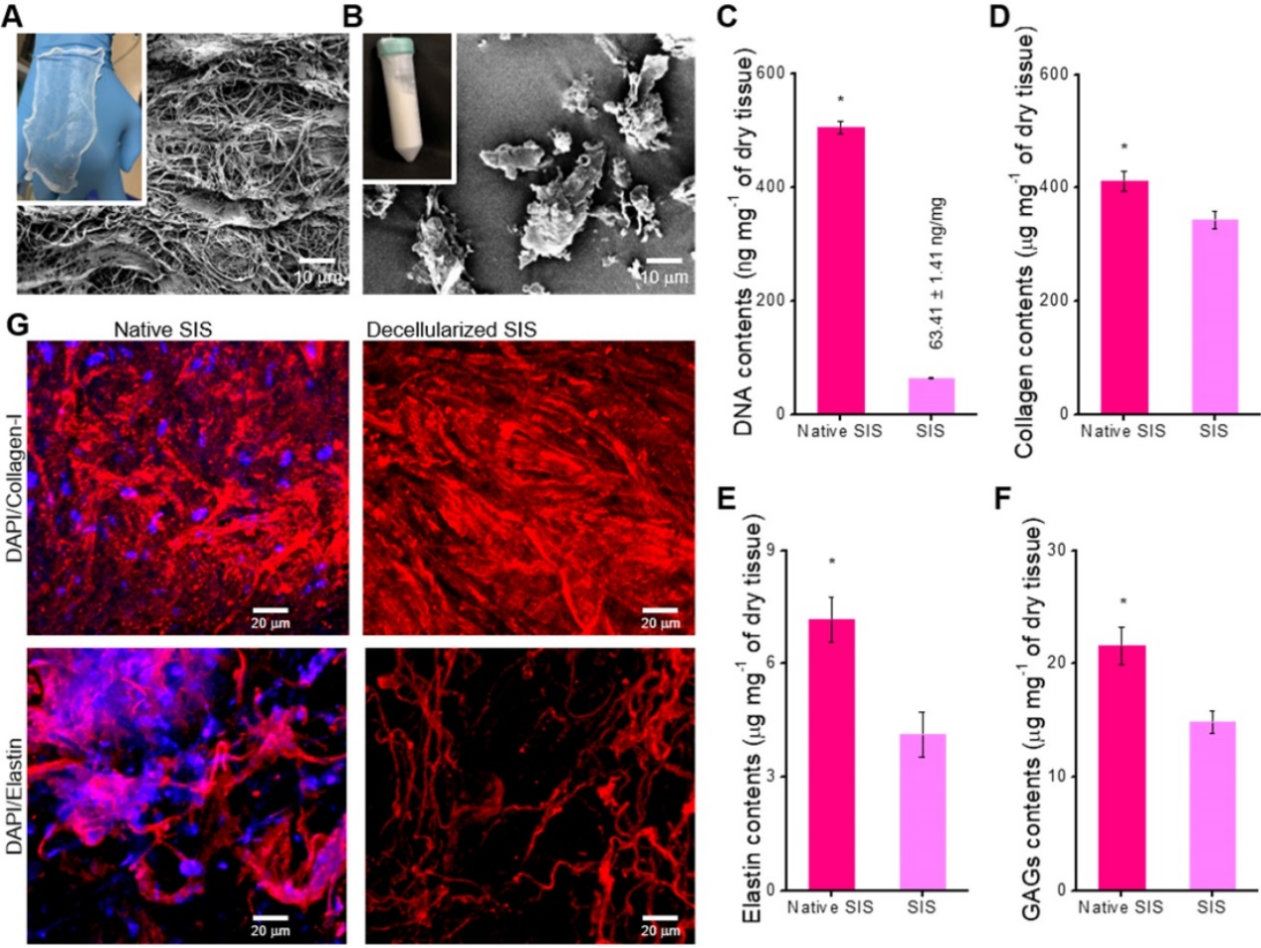
Optical and scanning electron microscopy (SEM) images of the decellularized small intestinal submucosa (SIS), (A) sheet and (B) powder. (C) DNA content, (D) collagen, (E) α-elastin, and (F) glycosaminoglycan (GAG) contents of native and decellularized SIS (n = 5, ^*^P < 0.5). (G) Immunofluorescence images (DAPI/collagen-type-I and DAPI/elastin) before and after decellularization.

The SIS has been extensively used as a natural ECM biomaterial for tissue regeneration because it has considerable biocompatibility and induces a significant level of biological activities 38. Here, the dECM biomaterial, SIS, was attained using the treatment to remove the component of the cells that can induce immune response 34. Figure 1A-B shows the optical microscope and SEM images of the finally fabricated SIS decellularized with 0.1% peracetic acid. The finally treated SIS appeared as a white, thin substance, and it was freeze-milled to obtain SIS powder. The SIS can be used as a bioactive component in collagen-bioink to obtain the intestinal scaffold; hence, after obtaining it, we measured the amounts of bioactive components, such as collagen, glycosaminoglycans (GAGs), and elastin. In addition, the DNA content (0.06 ± 0.001 μg/mg) was also observed quantitatively, as shown in Figure 1C. As shown in the results, the cell-component evoking an immune response was fully removed, whereas the extracellular proteins and polysaccharides were still resident in the SIS (Figure 1D-F). Figure 1G shows the immunofluorescence images of DAPI/collagen type-I and DAPI/elastin before and after the denaturalization treatment. In the images, the nuclei were well removed, whereas the proteins were well resident in the decellularized SIS.

**Figure 2:**
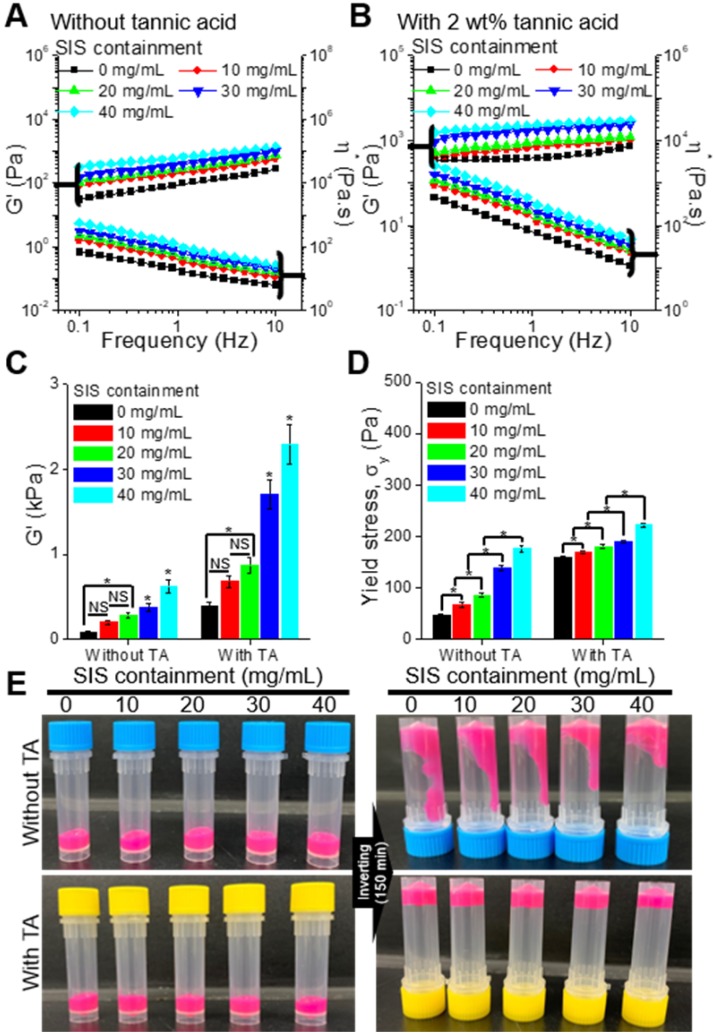
Storage modulus (G') and complex viscosity (η^*^) of the bioinks containing various concentrations of SIS (0, 10, 20, 30, and 40 mg/mL), (A) with and (B) without tannic acid (TA; 2 wt%). (C) G' of the bioinks at a frequency of 1 Hz. (D) Yield stress (σ_y_) of the bioinks. (E) Optical images showing the relative viscosity of the bioinks after mixing for 150 min.

To observe the effect of the SIS component on the rheological properties (storage modulus, G', complex viscosity, η^*^, and yield stress, σ_y_) of the bioinks, we used various bioinks that were obtained with the mixture of collagen (4 wt%) and various weight fractions (10, 20, 30, and 40 mg/mL) of SIS (Figure 2A). As the SIS component in the bioink increased, the modulus of the bioink increased gradually (Figure 2B, Figure S1(A)). In addition, to observe the effect of the crosslinking agent on the rheological properties, we measured the properties of the bioinks crosslinked with 2 wt% of TA (Figure 2B). As expected, the modulus and viscosity of the bioinks increased after the crosslinking with TA (Figure 2C, Figure S1(B)). The yield stress, which indicates whether the bioinks can be ejected through the nozzle and maintain the printed shape, was analyzed using the crossover shear stress value of the G' and G'' evaluated by a shear stress sweep (Figure S1(C,D)) 39, 40. The addition of SIS in the collagen-based bioink, which increased the bioink containment, and crosslinking with TA further reinforced the ability of structural maintenance (Figure 2D). Figure 2E presents optical images showing the relative viscosity of the bioinks after 150 min. The results were sufficiently coincident with those of the rheological measurement.

**Figure 3:**
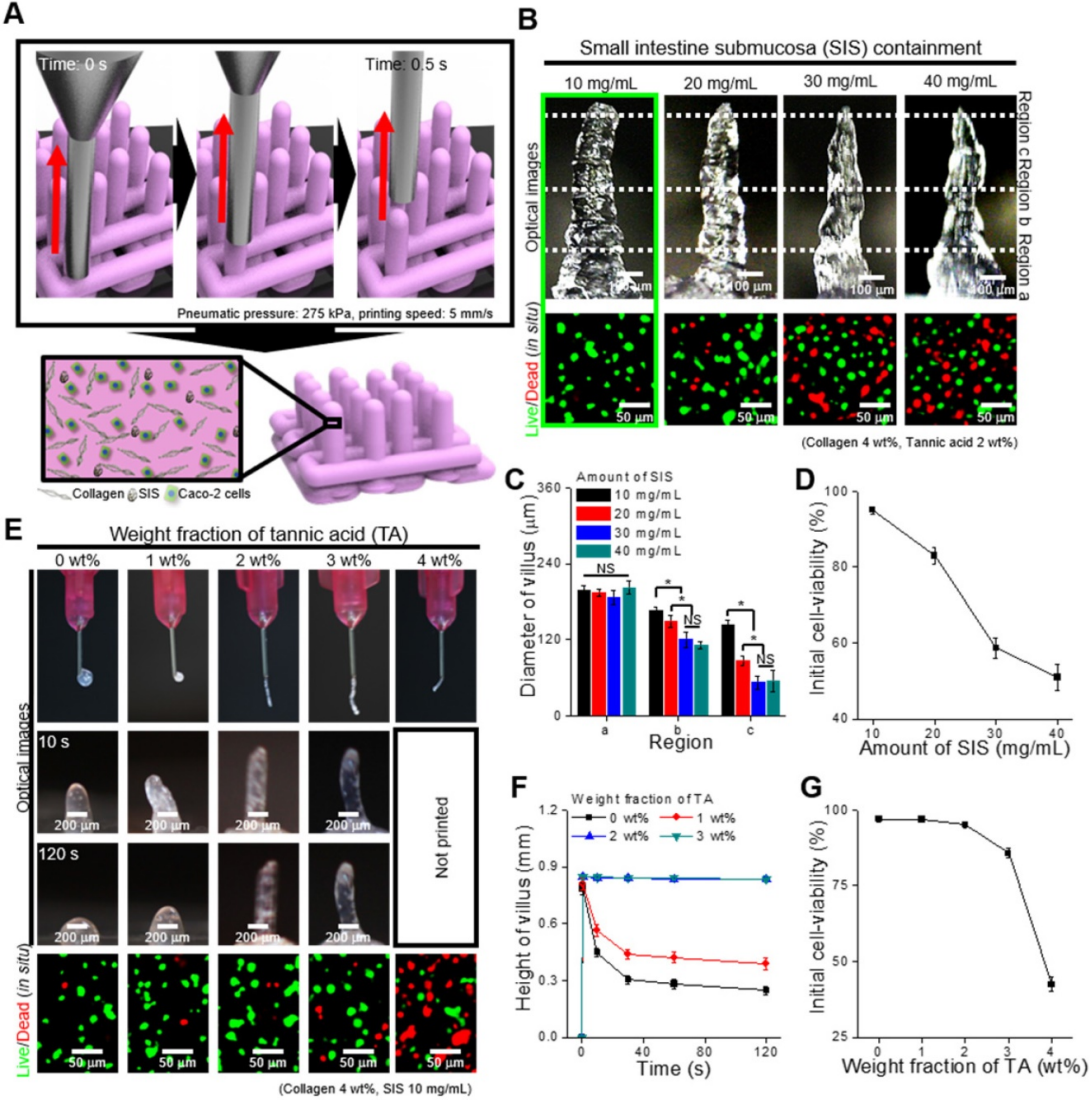
(A) Schematic of a vertically moved 3D printing process for fabricating Caco-2-laden 3D intestinal villi using collagen/SIS bioink. (B) Optical and live (green)/dead (red) images for various concentrations of the decellularized SIS (0, 10, 20, 30, and 40 mg/mL). (C) Diameter of the three different regions (regions a, b, and c) of the villus structures measured using the optical images, and (D) initial cell-viability calculated using the live/dead images for various SIS concentrations. (E) Optical images showing the nozzle and the villus structure after printing for 10 s and 120 s, and live (green)/dead (red) images for various weight fractions of TA (0, 1, 2, 3, and 4 wt%). (F) Height of the printed villus structure after various time points using the collagen/SIS bioink with various weight fractions of TA. (G) Initial cell-viability calculated using the live/dead images for various weight fractions of TA.

To determine the effect of SIS concentration on printability and cell viability after printing, we fixed the TA concentration (2 wt%) and printing conditions (moving speed: 5 mm/s, pneumatic pressure: 275 kPa, extrusion time: 0.5 s). Figure 3B shows optical images of the fabricated villus structure and live (green)/dead (red) images of the laden Caco-2 cells (5 × 106 cells/mL). The results show that the printed villus structure has a slightly different shape according to the weight fraction of SIS, determined by the measured dimension (regions a, b, and c) of the villus (Figure 3C), owing to the possibility of a non-homogeneous viscose region of the SIS phase. In addition, the cell viability of the printed villus with a high weight fraction of SIS (~ 20 mg/mL) was significantly low due to the high wall shear stress, evoked by the relative high viscosity, in the printing nozzle (Figure 3D). From the results, we can observe that the bioink with 10 mg/mL SIS showed stable structure formation and reasonable cell-viability (~ 90%) compared with others.

To observe the effect of TA concentration on the printability and cell viability, we used the bioink mixed with 10 mg/mL of SIS. Figure 3E shows the optical and live/dead images of the printed villus structure. A low concentration of TA (below 1 wt% of TA), which indicated a low crosslinking degree, induced a villus structure having insufficient mechanical stiffness, whereas a relatively high weight fraction of TA (over 4 wt% of TA) induced unsteady extrusion of the bioink due to the non-homogeneous flow, or high viscosity of the bioink with excessive yield stress 39, 40, which can be triggered by an excessively high degree of collagen crosslinked with the TA (Figure 3E-F). The results were correspondent with the rheological properties, in which increasement of the yield stress by the addition of TA enabled to maintain the structure after printing (Figure 3F, Figure S2). In addition, the cell viability was reasonably high (~ 90%) in the range below 2 wt% of TA in the bioinks (Figure 3G). Based on these results, the SIS and TA concentrations in the bioink were selected as 10 mg/mL and 2 wt% because of their tendency to induce mechanically stable villus formation and appropriate cell viability of the printed shape.

**Figure 4:**
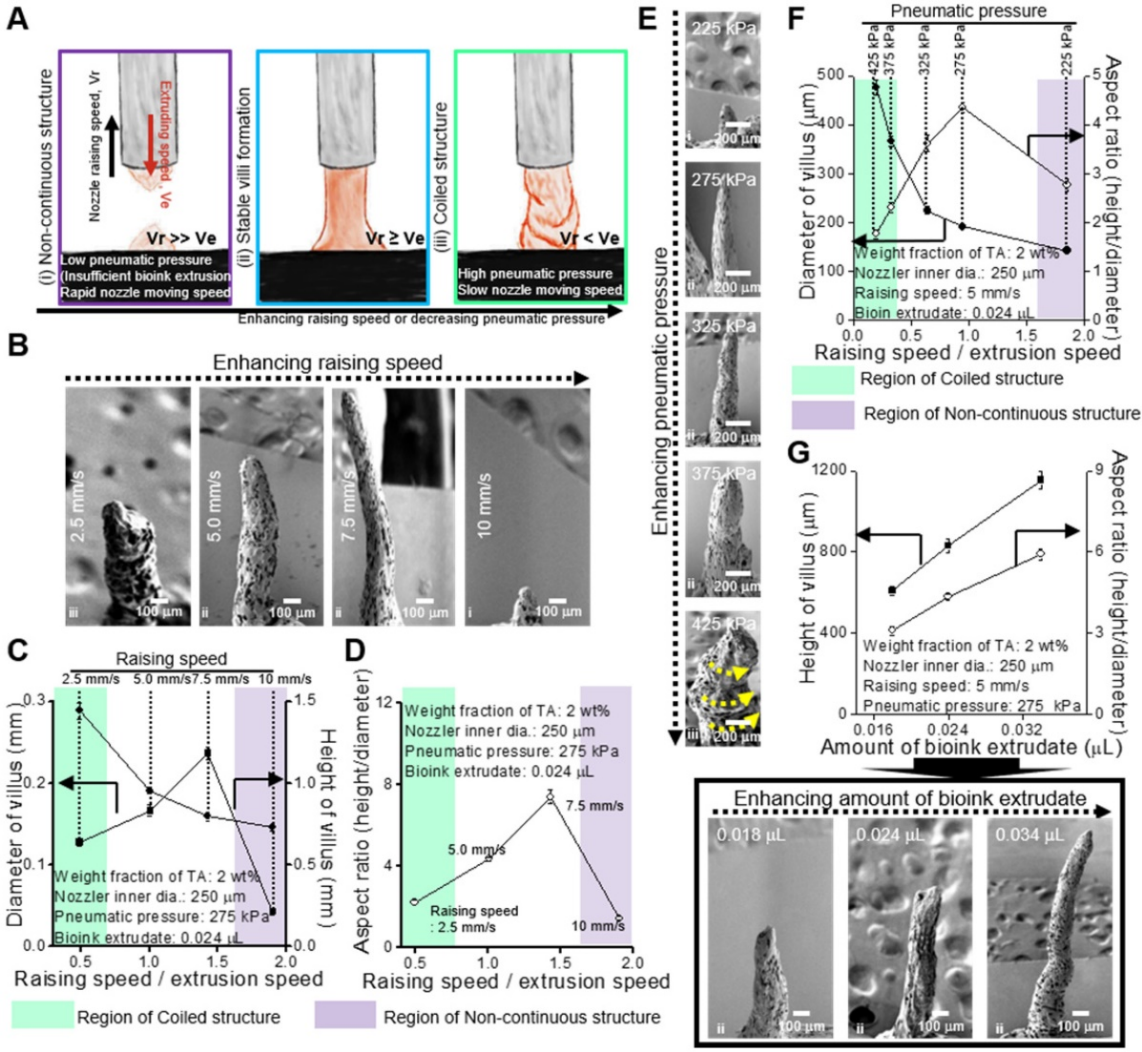
(A) Schematics showing various printed structure types ((i) non-continuous, (ii) stable, and (iii) coiled) using collagen (4 wt%)/SIS (10 mg/mL) bioink crosslinked with 2 wt% TA under different processing conditions. (B) SEM images showing the printed villus structures using various printing speeds on z-axis (2.5, 5, 7.5, and 10 mm/s) under a fixed condition (nozzle inner diameter (ID): 250 μm, extrusion time: 0.5 s, and pneumatic pressure: 275 kPa). (C, D) Processing diagrams demonstrating the fabricated 3D geometries vs. the printing speed. (E) SEM images of the printed villus structures for various pneumatic pressures (225, 275, 325, 375, and 425 kPa) under a fixed condition (nozzle inner diam. (ID): 250 μm, extrusion time: 0.5 s and printing speed: 0.5 mm/s). (F) Process diagram demonstrating the applied pneumatic pressure. (G) The Fabricated 3D geometries vs. the amount of bioink extrudate and SEM images of the printed villus structures for various amount of bioink extrudate (0.018, 0.024, and 0.034 μL).

To obtain stable formation of the villus structure, we should select stable processing conditions for the printing parameters, such as dispensing speed, printing time, and pneumatic pressure. Under different processing conditions, three types of printed structures are observed (Figure 4A): (i) non-continuous structure, (ii) stable villus formation, and (iii) coiled structure. Figure 4B-G presents the processing diagrams of the collagen/SIS bioinks with respect to the printing speed on the z-axis, printing time, and pneumatic pressure.

Figure 4B shows the SEM images of a fabricated villus structure for various printing speeds (raising speed) in relation to the z-axis at fixed processing conditions (nozzle diameter: 250 μm, pneumatic pressure: 275 kPa, and extrusion time: 0.5 s). As shown in the images, as the raising speed was increased, the height of the villus was gradually increased, but low (below 2.5 mm/s) or excessively high raising speeds (over 10 mm/s) induced unstable villus structure formations, such as a coiling structure at 2.5 mm/s or a non-continuous structure at 10 mm/s. The coiling effect can be generally found in an extrusion process as the nozzle-to-working stage distance (falling height) reaches a certain falling height, and the phenomenon can be highly dependent on the flow rate and viscosity of the extrudate 41-45. The high extruding speed (volume flow rate of bioink) relative to the printing speed could cause the over-deposition of the filament or liquid-based inks and induce the coiling effect 44, 45. Likewise, at 2.5 mm/s of the raising speed, the over-deposition of collagen/SIS bioink induced the coiled structure due to the high volume flow rate. From the analysis of the SEM images, we can observe that, as the speed increased, the diameter and height of the villus were decreased and increased, respectively, until a certain range, and therefore, a maximum aspect ratio (height/diameter = 8) could be achieved (Figure 4C-D).

In addition, the pneumatic pressure (or volume flow rate) can directly affect the formation of the villus. We measured the structure formation for various pneumatic pressures at constant raising speed (5 mm/s) and extrusion time (0.5 s) (Figure 4E) and found a reasonable range to fabricate a stable finger-like villus structure (Figure 4F). Interestingly, we also observed the coiling effect under a high pneumatic pressure (425 kPa).

To observe the effect of printing time on the formation of the villus structure, we used various amounts of bioink extrudate (0.018-0.034 μL) at a constant raising speed, 5 mm/s, and the same pneumatic pressure (Figure 4G). As expected, the height of villus was gradually increased with increasing the amount of bioink extrudate (Figure 4G).

Using the processing diagrams, we can select an appropriate processing condition (pneumatic pressure: 275 kPa, printing speed: 5.0 mm/s, extrusion time: 0.5 s) for the villus structure (height: 831.1 ± 36.2 μm, aspect ratio: 4.4 ± 0.1) without compromising cell viability.

### Fabrication of CLIV-C and CLIV-CS villus structure

**Figure 5:**
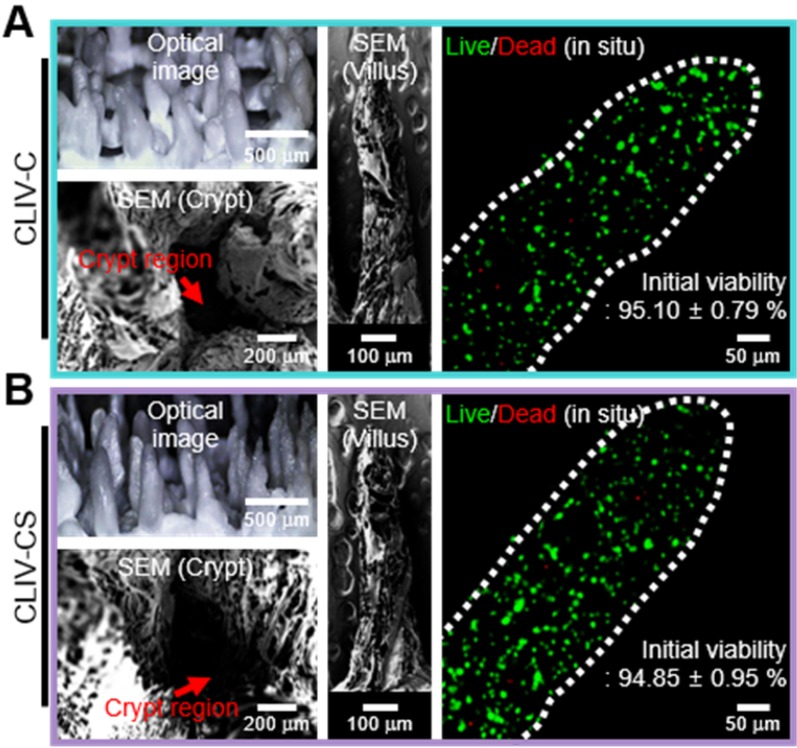
(A) Optical and SEM images and (B) cell viability of the two fabricated villus structures using the bioinks.

To detect the effect of the SIS component laden in the villus structure on the cellular activities, we fabricated two villus structures, (1) Caco2-laden intestinal villi printed with a collagen bioink (CLIV-C) as a control and (2) Caco2-laden collagen/SIS villus structure (CLIV-CS) as an experimental group. The fabrication material and process parameters for obtaining a stable villus structure are summarized in Table 2. In Figure 5A, optical and SEM images of the two fabricated villus structures using the bioinks are shown. As shown in the images, the cell-laden villus structure (831.1 ± 36.2 μm in height and with an aspect ratio of 4.4 ± 0.1) was a construct similar to that of the human gastrointestinal tract (721.4 ± 20.0 μm in height and with an aspect ratio of 4.7 ± 0.3 46). In addition, the Caco-2 cells were well spread in both villus structures, and reasonable cell viability was obtained (Figure 5B). The results demonstrate that the bioprinting process is a completely stable and safe process for the laden cells, to fabricate the intestinal 3D villus model.

### *In vitro* cellular activities of CLIV-C and CLIV-CS villus structure

**Figure 6:**
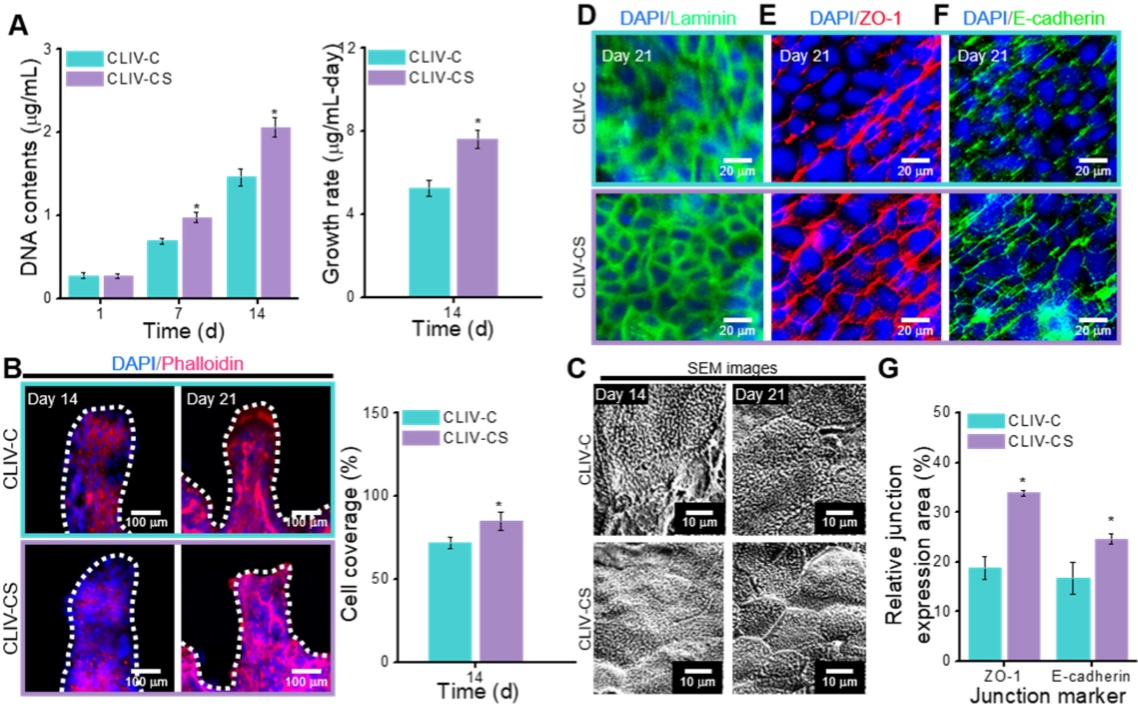
(A) DNA content (1, 7, and 14 days of culture) and growth rate (14 days of culture) for the Caco-2-laden 3D intestinal villi with collagen (CLIV-C) and collagen/SIS (CLIV-CS). (B) DAPI (blue)/phalloidin (red) images (14 and 21 days of culture) and cell coverage area (%) of cytoskeleton calculated using the phalloidin images at 14 days for the 3D models. (C) SEM images showing the microvilli on the surface of the scaffolds. The immunofluorescence images show the formation of (D) basement membrane (laminin, green), (E) tight junction (ZO-1, red), and (F) adherent junction (E-cadherin, green) for the Caco-2 cells cultured in the 3D intestinal models at 21 days of culture. (G) Relative ZO-1 and E-cadherin expression area for the 3D models calculated using the immunofluorescence images at 21 days of culture.

In this section, we compare the cellular activities, such as cell proliferation and immunofluorescence images showing cytoskeleton and cell-to-cell interaction, of the control group with those of the experimental group. To evaluate the cell proliferation, the DNA content, determined by picogreen assay, was measured for the control and experimental groups (Figure 6A). As shown in the results, the Caco-2 cells laden in the CLIV-CS proliferated more actively than those in the CLIV-C at 7 and 14 days. Moreover, the proliferation rate at 14 days for the experimental group was significantly higher than that for the control. The results were well validated with the DAPI (blue)/Phalloidin (red) results at 14 days, shown in Figure 6B. Unlike the CLIV-CS scaffold, the cultured cells in the CLIV-C scaffold displayed some uneven growth on the scaffold surface, indicating that the conflux of the epithelial cell-layer was not completely uniform. However, the Caco-2 cells at 21 days adequately covered both structures, like a mono-layer. In addition, the Caco-2-laden mesh structures without villi were fabricated using collagen (CLM-C) and collagen/SIS (CLM-CS). The cells laden in CLM-CS showed significant increase in cellular growth compared to that in CLM-C (Figure S3(A-C)). However, the cells were proliferated more rapidly in CLIV-C and CLIV-CS, which contain villus structures (Figure S3(B-C)). The results are correspondent with the previous study of De Gregorio et al. 47 that the villus structures provided a favorable microenvironment to accelerate the growth of Caco-2 cells. Figure 6C shows SEM images of the microvilli on the scaffold surfaces at 14 and 21 days. As shown in the result, the microvilli, which are known as the brush border, were formed much more rapidly and homogeneously on the surface of the CLIV-CS than on that of the control. Likewise, the cells in the CLM-CS formed brush border structures more rapidly and homogeneously compared to those in the CLM-C (Figure S3(C)).

To obtain a proper *in vitro* epithelial model, the cell confluence and barrier integrity are important design parameters 3. To observe the confluent epithelial layer, we assessed the stained laminin after 21 days of cell culture in the control and experimental groups because the laminin can provide an attachment framework for the epithelial cells. The immunofluorescence result demonstrated a confluent epithelial layer on both the control and experimental groups (Figure 6D). However, the development of cell-to-cell adhesion for the structures stained with the junction marker (ZO-1 and E-cadherin) was different. In Figure 6E-F, the main junction proteins (ZO-1 and E-cadherin) on Caco-2 cells cultured for 21 days showed a distinctive cobblestone mark shaped in the layer of the epithelium on the CLIV-C and CLIV-CS scaffolds. However, as shown in the images, the tight (ZO-1) and adherent (E-cadherin) junctions in the CLIV-CS were more homogeneously and clearly developed in the cells than those in the control (Figure 6G). In the case of the mesh structures without villus structures (CLM-CS and CLM-C), the laminin was well expressed in the cells in both mesh structures, while the tight and adherent junctions were developed more in the CLM-CS compared to those in the CLM-C (Figure S3(D-E)). The cell confluence and barrier integrity results demonstrated that the biological molecules in the SIS support the Caco-2 cells to form the matured epithelium layer 48.

**Figure 7:**
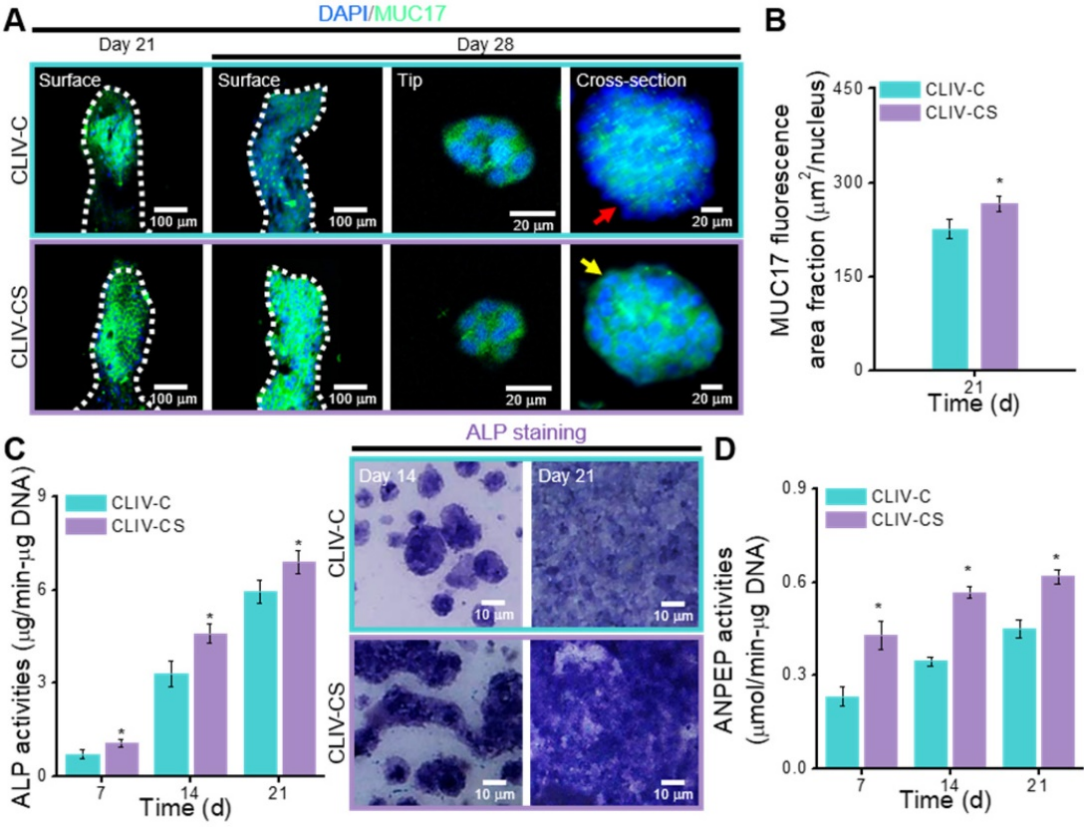
(A) Surface, tip, and cross-sectional DAPI (blue))/MUC17 (green) images of the Caco-2 cells in the CLIV-C and CLIV-CS after 21 and 28 days of culture. (B) MUC17 area fraction of CLIV-C and CLIV-CS calculated using the MUC17 images after 21 days of culture. Enzymatic results, (C) ALP activities (7, 14, and 21 days of culture) and ALP staining optical images, and (D) ANPEP activities (7, 14, and 21 days of culture) in CLIV-C and CLIV-CS.

In the intestinal mucosal surface, mucins engage in protection by blocking noxious interactions of epithelial cells with pathogens by providing a physiochemical barrier 49. To observe the expression on the surface of intestinal epithelia, we measured the MUC17, which is one of the indicators that shows the membrane-bound mucins 4. Figure 7A shows the MUC17 on the structures cultured for 21 and 28 days (Figure S3(F)). The expressions of MUC17 at 21 and 28 days were significantly different between the structures fabricated using collagen and collagen/SIS bioinks (Figure 7B, Figure S3(G)).

To detect the differentiation of epithelial cells on the CLIV-C and CLIV-CS scaffolds, the activity of ALP, which is a brush-border enzyme 50 that indicates the expression of an enterocyte differentiation marker, was evaluated, as shown in Figure 7C. Based on the qualitative and quantitative results, the ALP activity was significantly higher in the experimental scaffold than in the control. In addition, ANPEP, which is a digestive enzyme catalyzing the cleavage of proteins 51, was measured, as shown in Figure 7D. The ANPEP activity in the CLIV-CS was significantly higher than that in the CLIV-C throughout the culture period. In the case of the mesh models without villus structures, the ALP and ANPEP activities in the CLM-CS were significantly enhanced compared to those in the CLM-C (Figure S3(H-I)). This phenomenon occurred because the bioactive components of the SIS can adequately affect the proliferation and even differentiation of the Caco-2 cells. Furthermore, the effects of the bioactive cues on the differentiation of the epithelial cells were greater than the existence of villus structures (Figure S3(H-I)).

**Figure 8:**
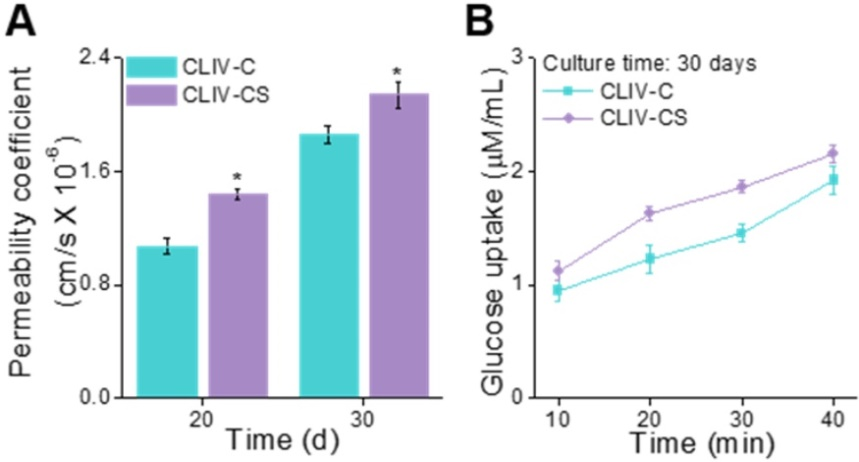
(A) Permeability coefficient (20 and 30 days of cell culture) and (B) glucose uptake ability (30 days of culture) of the scaffolds, CLIV-C, and CLIV-CS.

To observe the permeability coefficient and the glucose uptake ability, the CLIV-C and CLIV-CS scaffolds, which were cultured over 20 and 30 days, were used. In Figure 8A-B, the permeability coefficient and glucose uptake (30 days of cell-culture) are depicted, and the CLIV-CS comprising dense and homogeneous brush border structures shows significantly higher permeability and glucose uptake, although both scaffolds have similar 3D protruding shapes and aspect ratios 52, 53. In addition, the absorption capacity was improved owing to the existence of villus structures with broader surface area (Figure S3(J-K)) 10, 36. The results indicate that the SIS-biocomponents laden in the collagen bioink and intestine-mimetic 3D structure clearly induced the development of the epithelial barrier in a much more similar manner to that of the human small intestine, this mimicking it, compared with the pure collagen structure.

In this study, we observed the effect of SIS components on the fabrication of the finger-like villus structure and the maturation of Caco-2 epithelial cells because the SIS has been widely applied to various tissue-engineering materials. To achieve the proper geometrical dimensions of the human villus structure and a sufficiently high cell viability in the printed structure, various concentrations of the SIS and crosslinking agent were used, and the most appropriate concentrations were selected. After fabricating the unique structure using the modified bioprinting process, the Caco-2-laden scaffolds were cultured for up to 30 days to assess the cell proliferation and differentiation, including the formation of the barrier junction and the development of mucins. A significant increase in the cell-growth rate and a high degree of differentiation were observed in the groups using the SIS component compared with the pure collagen cell-laden scaffold. Based on the *in vitro* cellular responses and the glucose uptake ability, we can confirm that the SIS-component can be helpful in the construction of the epithelial layer of the intestine. However, in this study, we were unable to reveal which components of the SIS directly contributed to the formation of the epithelial layer. Further investigation on the relation between the SIS component and the development of the epithelial layer will be performed in our future studies.

### Application of fabrication system for 3D intestinal model consisting of epithelium and capillaries

**Figure 9:**
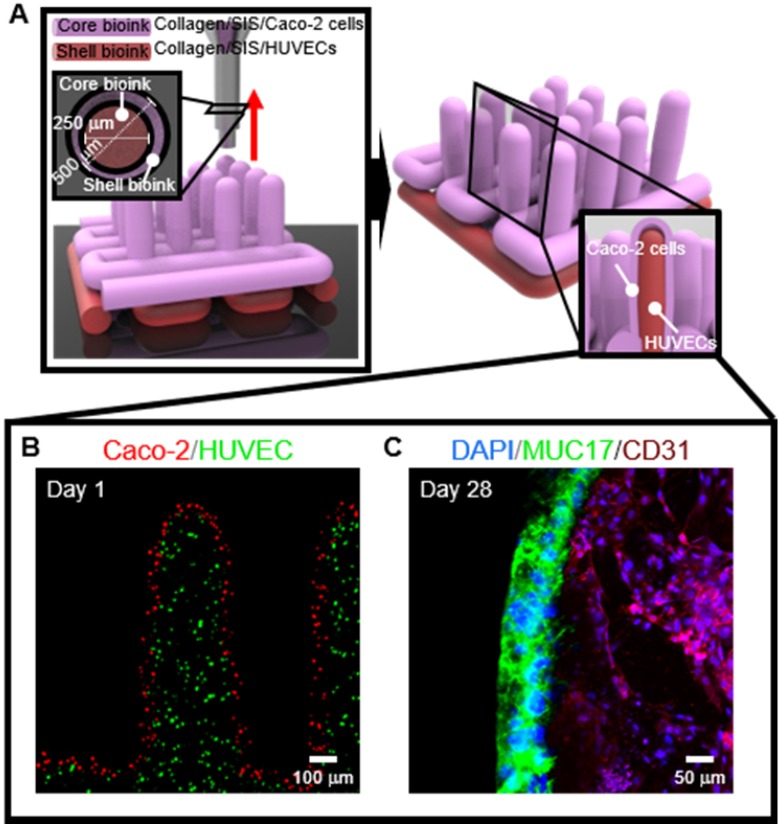
(A) Schematic showing a 3D printing process supplemented with core/shell nozzle for fabricating 3D intestinal model with epithelium and capillaries using collagen/SIS bioinks. (B) Cell tracker image showing the fabricated 3D model consisting of epithelium region with Caco-2 cells (red) and capillary region with HUVECs (green) at 1 day of culture. (C) DAPI (blue)/MUC17 (green)/CD31 (purple) image showing the matured epithelium monolayer and capillaries at 28 days of culture.

To obtain the 3D intestinal model containing an epithelium monolayer and microvascular structures, HUVEC-laden (core bioink) and Caco-2-laden (shell bioink) collagen/SIS bioinks were printed by using 3D cell-printing system connected with core/shell nozzle, as shown in Figure 9A. As shown in the cell-tracker image of Figure 9B, two cell types were located distinctly as HUVECs (green) in the core region and Caco-2 cells (red) in the shell region. After 28 days of culture, the cultured model was stained with MUC17 for Caco-2 cells and cluster of differentiation 31 (CD31), which is a protein encoded by the platelet endothelial cell adhesion molecule (PECAM1) gene of human 54, for HUVECs. Both of MUC17 and CD31 were well expressed from the cultured Caco-2 cells and HUVECs (Figure 9C). Furthermore, the Caco-2 cells showed an epithelial barrier in a monolayered structure in the shell region, and the HUVECs formed capillary networks in the core region, as shown in the cross-sectional fluorescence image (Figure 9C). The results indicate that the collagen/SIS bioink and cell-printing system could be an efficient tool for fabricating a stable intestinal model mimicking the complex human intestine anatomically. Further investigation on the development of biomimetic 3D intestinal models containing complex structures, including epithelium, capillaries, and lacteals will be performed in our future studies 46.

## Conclusions

In this study, to obtain a 3D biomimetic villus model, a bioprinting process using a bioink consisting of epithelial cells and a collagen/SIS biocomponent was used. The unique process allowed the use of various material and processing conditions (SIS concentration, weight fraction of TA, printing speed, printing time, and pneumatic pressure) in designing the biomimetic villus structure. The epithelial cell-laden collagen/SIS villi showed significant cell proliferation (1.2-fold) and demonstrated meaningful results for the various cellular activities, such as the expression of tight-junction proteins (ZO-1 and E-cadherin), ALP and ANPEP activities, MUC17 expression, and the permeability coefficient and the glucose uptake ability, compared with the pure 3D collagen villus structure. By using the epithelial villus model, we will attempt to measure absorption screening, host microbial work, and other activities in the near future.

## Supplementary Material

Supplementary figures.

## Figures and Tables

**Table 1:** Fabrication processes for 3D intestinal models.

Matrix material	Cell-type	Process to obtain 3D structure	Ref.
Collagen	Caco-2	Replica molding process	^4^
Collagen/PEG	Caco-2	laser ablation/sacrificial molding	^7^
PLGA	Caco-2	Replica molding process	^8^
Collagen	Caco-2	Direct 3D cell-printing	^9^
PDMS/Collagen/Matrigel	Caco-2	Cell self-assemble in a microchannel	^10^

PEG: polyethylene glycol / PLGA: poly(lactic-*co*-glycolic acid) / PDMS: polydimethylsiloxane.

**Table 2:** Bioink compositions and process parameters for fabricating CLIV-C and CLIV-CS scaffolds.

	Materials			Process parameters		
	Collagen (wt%)	SIS (mg/mL)	TA (wt%)	Printing speed (mm/s)	Pneumatic pressure (kPa)	Extrusion time (s)
CLIV-C	4	0	2	5.0	250	0.5
CLIV-CS	4	10	2	5.0	275	0.5
