# Supplementary material for: An intestinal model with a finger-like villus structure fabricated using a bioprinting process and collagen/SIS-based cell-laden bioink
Source: Theranostics. 2020 Jan 22;10(6):2495–508. doi: 10.7150/thno.41225 (PMC7052892; doi:10.7150/thno.41225)
Supplement: Supplementary file 1 — Supplementary figures. [file thnov10p2495s1.pdf]

Supplementary Information

An intestinal model with a finger-like villus structure fabricated using a bioprinting process and collagen/SIS-based cell-laden bioink

WonJin Kim and Geun Hyung Kim*

Department of Biomechatronic Engineering, College of Biotechnology and Bioengineering, Sungkyunkwan University (SKKU), Suwon, 16419, Republic of Korea

Running title: Bioprinted 3D intestinal model

***Corresponding author**

GeunHyung Kim, Ph.D
Professor
Department of Biomechatronic Engineering, College of Biotechnology and Bioengineering, Sungkyunkwan University (SKKU), Suwon, South Korea.
Email: gkimbme@skku.edu, Tel.: +82-31-290-7828.

Supporting figures

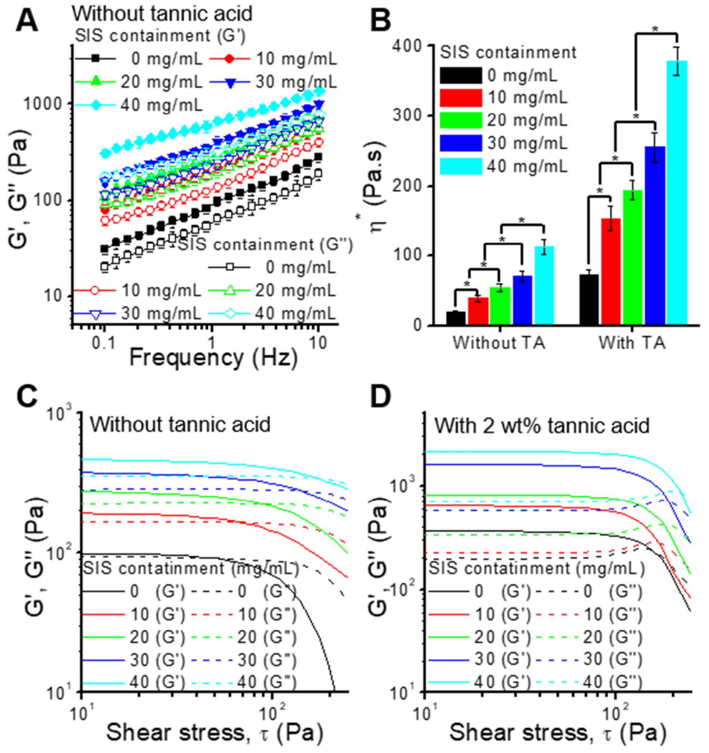

Figure S1. (A) Storage modulus (G') and loss modulus (G'') of the bioinks containing various concentrations of SIS (0, 10, 20, 30, and 40 mg/mL), without tannic acid (TA). (B) G' of the bioinks with and without 2 wt% TA at a frequency of 1 Hz. G' and G'' of the bioinks containing various concentrations of SIS (0, 10, 20, 30, and 40 mg/mL), (C) with and (D) without crosslinking agent (TA; 2 wt%).

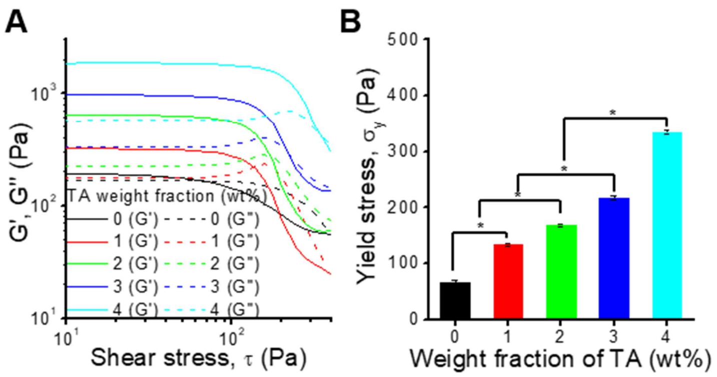

Figure S2. (A) Storage modulus (G') and loss modulus (G'') of the bioinks containing 10 mg/mL SIS, with various weight fractions of crosslinking agent (TA; 0, 1, 2, 3, and 4 wt%). (B) Yield stress (σ_y) of the bioinks.

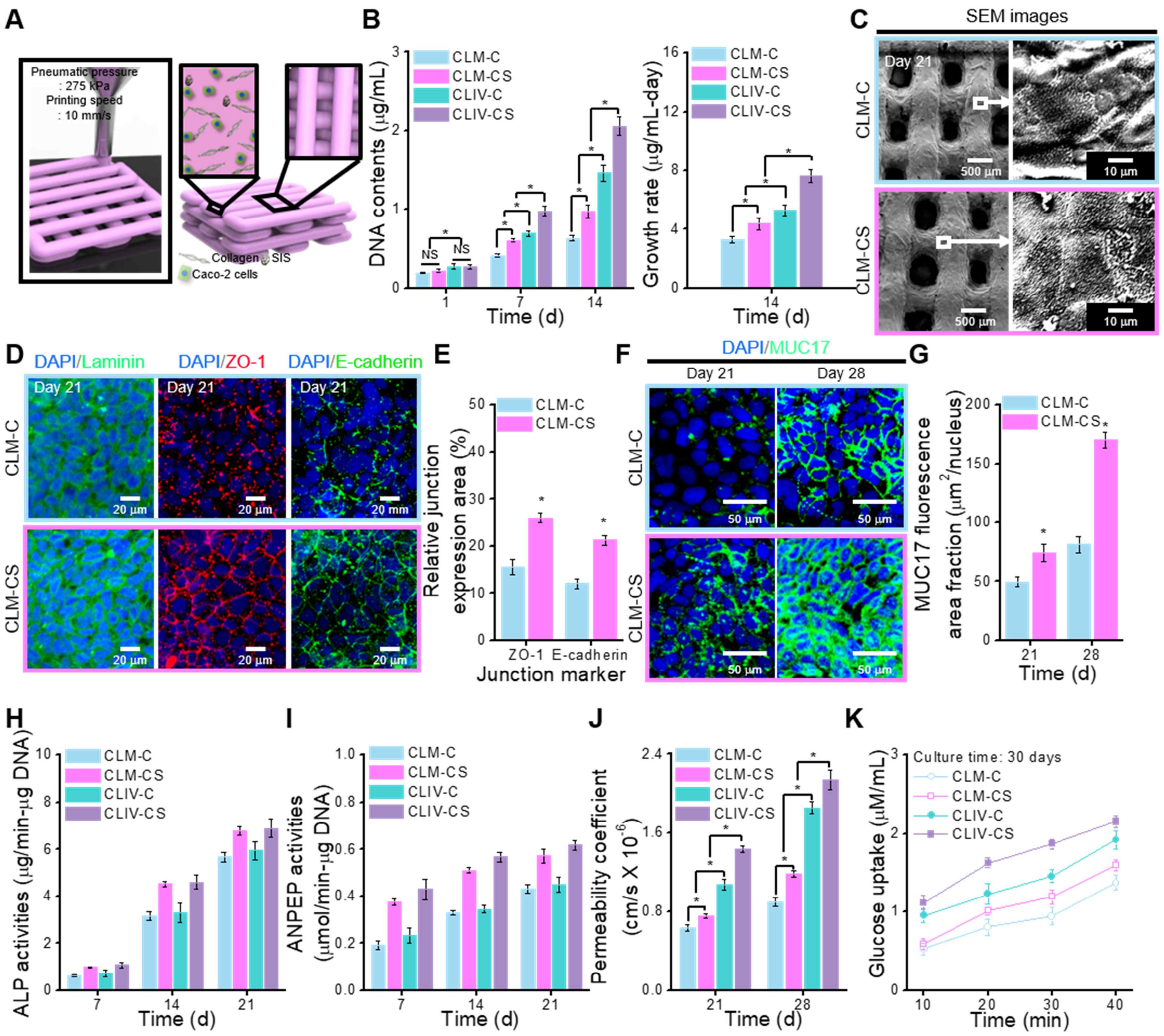

Figure S3. (A) Schematic showing a 3D printing process for fabricating Caco-2-laden 3D mesh model without villus-structures using collagen/SIS bioink. (B) DNA content (1, 7, and 14 days of culture) and growth rate (14 days of culture) for the Caco-2-laden models with (3D intestinal models; CLIV-C and CLIV-CS) and without (intestinal mesh models; CLM-C and CLM-CS) villus-structures printed using collagen and collagen/SIS bioinks. (C) SEM images for the intestinal mesh models without villus-structures (CLM-C and CLM-CS) showing the brush border formation at 21 days of culture. (D) The immunofluorescence images show the formation of basement membrane (laminin, green), tight junction (ZO-1, red), and adherent junction (E-cadherin, green) for the Caco-2 cells in the mesh models at 21 days of culture. (E) Relative junction proteins expression area for the mesh models calculated using the ZO-1 and E-cadherin images. (F) DAPI (blue))/MUC17 (green) images of the Caco-2 cells in the CLM-C and CLM-CS after 21 and 28 days of culture. (G) MUC17 area fraction of the mesh models calculated using the MUC17 images. Enzymatic results, (H) ALP activities and (I) ANPEP activities for the 3D intestinal models and intestinal mesh models after 7, 14, and 21 days of culture. (J) Permeability coefficient (20 and 30 days of cell culture) and (K) glucose uptake ability (30 days of culture) of 3D intestinal models and intestinal mesh models.
